# HOXB4 Mis-Regulation Induced by Microcystin-LR and Correlated With Immune Infiltration Is Unfavorable to Colorectal Cancer Prognosis

**DOI:** 10.3389/fonc.2022.803493

**Published:** 2022-02-08

**Authors:** Lingqiao Wang, Huidong Jin, Yi Zeng, Yao Tan, Jia Wang, Wenjuan Fu, Weiyan Chen, Ke Cui, Zhiqun Qiu, Ziyuan Zhou

**Affiliations:** ^1^ Department of Environmental Health, College of Preventive Medicine, Army Medical University (Third Military Medical University), Chongqing, China; ^2^ Institute of Pathology and Southwest Cancer Center, Southwest Hospital, Army Medical University (Third Military Medical University), Chongqing, China

**Keywords:** microcystin-LR, HOXB4, colorectal cancer, prognosis, tumor-infiltrating immune cells

## Abstract

Microcystin-LR (MC-LR) exists widely in polluted food and water in humid and warm areas, and facilitates the progression of colorectal cancer (CRC). However, the molecular mechanism associated with the MC-LR-induced CRC progression remains elusive. The purpose of this study is to explore the role of the hub genes associated with MC-LR-induced CRC development at the molecular, cellular and clinical levels through bioinformatics and traditional experiments. By utilizing R, we screened and investigated the differentially expressed genes (DEGs) between the MC-LR and the control groups with the GEO, in which, HOXB4 highly expressed in MC-LR-treated group was identified and further explored as a hub gene. With the aid of TCGA, GEPIA, HPA, UALCAN, Cistrome, and TIMER, the increased mRNA and protein levels of HOXB4 in CRC tissue were found to be positively associated with high tumor stage and poor prognosis, and were linked to immune infiltration, especially tumor-associated macrophages and cancer-associated fibroblasts. Cox regression analysis and nomogram prediction model indicated that high HOXB4 expression was correlated to poor survival probability. To elucidate the mechanism of high HOXB4 expression induced by MC-LR, we overlapped the genes involved in the MC-LR-mediated CRC pathways and the HOXB4-correlated transcription genes. Importantly, C-myc instead of PPARG and RUNX1 promoted the high expression of HOXB4 through experiment validation, and was identified as a key target gene. Interestingly, C-myc was up-regulated by HOXB4 and maintained cell cycle progression. In addition, MC-LR was proved to up-regulate HOXB4 expression, thus promoting proliferation and migration of Caco2 cells and driving the cell cycle progression. In conclusion, MC-LR might accelerate CRC progression. In the process, MC-LR induced C-myc augmentation elevates the high expression of HOXB4 through increasing the S phase cell proportion to enhance Caco2 cell proliferation. Therefore, HOXB4 might be considered as a potential prognostic biomarker for CRC.

## Introduction

Microcystins (MCs) are the largest and most diverse group of cyanotoxins (1). Of over 270 structural variants of MCs, microcystin-LR (MC-LR) is the most studied congener and considered as one of the most potent cyanobacterial toxins based on previous toxicity studies (1, 2). In particular, regular water treatment technology, such as filtration and chlorination disinfection, and conventional heating (100°C, 30min) cannot effectively remove MCs (3), and therefore chronic environmental exposure to MCs, either directly through drinking water or indirectly through food-chain and dermal absorption, has been a health concern worldwide.

Colorectal cancer (CRC) is the third most common cancer and the second leading cause of cancer-related mortality worldwide (4). Generally, family disease history (5), diet structure, living habits (6), and chronic inflammatory irritation (5) are recognized as causes of CRC. But recent epidemiological surveys reveal that low-dose and long-term exposure to MC-LR is effectively associated with CRC (7). Animal studies also provided evidence of effects of MC-LR on CRC progression (8, 9). Previous studies on the toxicity of MC-LR mainly concentrate on its impact on liver and kidney (10), but its effect on the gut, which is the primary target organ for MC-LR from both direct (drinking water) and indirect (food chain) exposure routes, is less studied (11). Considering the relatively poor overall prognosis of CRC, the mechanism linking MC-LR exposure to CRC is worth attention. To identify specific molecular markers for effects of MC-LR exposure on CRC progression may help understanding MC-LR-mediated carcinogenic activity and be in favor of CRC treatment.

Homeobox B4 (HOXB4) belongs to the homeobox (HOX) family that possesses transcription factor activity and is essential for stem cell self-renewal and tumorigenesis (12). It has been reported that HOXB4 participates in the advancement of ovarian cancer, as well as lung, prostate, and breast cancer (13). A previous report has shown that elevated HOXB4 expression facilitates the ovarian cancer progression *via* DHDDS (14). However, HOXB4 attenuates the tumorigenesis of cervical cancer cells by declining the Wnt/β-catenin signaling pathway activity (13). Bhatlekar, S. et al. reported that HOX gene overexpression signature for the normal human colonic crypt cell niche parallels stem cell overpopulation during colon tumorigenesis (15, 16). Meanwhile, the high expression of HOXB7, which is a homologous cluster with HOXB4, is considered to be a biomarker of the clinicopathological characteristics and poor prognosis in CRC dependent on activation of the PI3K/AKT pathway (17). However, the role of HOXB4 expression in CRC still remains unclear based on clinical big data.

In this work, microarray data analyses and experiment validation were implemented to comprehensively determine the pathways and hub genes that most likely participated in MC-LR-induced CRC progression. Our findings demonstrated that cell cycle progression was dependent on the promotion of C-myc, and that C-myc was regulated by MC-LR-induced high HOXB4 expression. Therefore, MC-LR enhanced CRC cell proliferation. Moreover, HOXB4 was linked to poor prognosis in CRC after covering a group of factors including gene expression, survival status, clinical-pathological parameters, immune infiltration, the biological function, and relevant cellular pathway.

## Materials and Methods

### Microarray Data

The gene expression profiles of MC-LR-exposed CRC cells by using GSE29861 dataset were obtained from GEO database (18). The GSE29861 dataset, based on Agilent GPL4133 platform (Agilent-014850 Whole Human Genome Microarray 4x44K G4112F), contained 9 samples of Caco-2 cells treated with MC-LR at concentrations of 0 μM and 5 μM for 24 h (19).

### Identification of the Differentially Expressed Genes (DEGs)

The normalized expression matrix of microarray data was downloaded from the GSE29861 dataset, and the probes corresponding to multiple molecules were then removed. The “limma” package of R software was used to identify DEGs across experimental condition, and the significant DEGs with *P*<0.05 and [logFC]>1.0 were considered as the cut-off criteria. The heatmap, UMAP, volcano plot and box plot were conducted by using “Complex Heatmap”, “umap” and “ggplot2” packages of R software.

### Gene Expression Analysis and the Human Protein Atlas

We utilized The Cancer Genome Atlas (TCGA) project (https://genome-cancer.ucsc.edu/) to analyze the expression level of HOXB4 in colon adenocarcinoma (COAD), as well as its expression level of messenger RNA (mRNA). To obtain the data on protein immunohistochemical analysis of tumor tissue and normal human tissue, the Human Protein Atlas online website (https://www.proteinatlas.org/) was used. Besides, the online pathology database provided protein-specific expression and location information for 48 normal tissue samples, 20 typical categories of cancer, 47 cell lines and 12 blood cells.

### Gene Ontology (GO) Analysis and Kyoto Encyclopedia of Genes and Genomes (KEGG) Pathway Enrichment Analysis

The GO project is a widely-used effective method for annotating genes and gene products and identifying characteristic biological attributes for high-throughput genome or transcriptome data. KEGG is a knowledge base for linking genomic information with higher-order functional information and systematic analysis of gene function (20). DAVID (https://david.ncifcrf.gov/) is a crucial foundation for the success of any high-throughput gene annotation, integrated discovery function and visualization analysis, which provides gene biological meaning for analyzing the function and pathway enrichment of DEGs (21). The KEGG pathway analysis, the GO enrichment analysis and the functional level analysis of the DEGs were performed by adopting the DAVID. The *P*-value <0.05 was considered statistically significant.

### Gene Expression Profiling Interactive Analysis (GEPIA) and UALCAN Database Analysis

To verify the prognostic values of HOXB4 in COAD patients, we obtained the significant data of HOXB4 on overall survival (OS) and disease free survival (RFS) of tumors derived from TCGA analyzed with GEPIA2 (test) and UALCAN (http://ualcan.path.uab.edu/analysis.html) online tools, which are newly developed web-based tools providing key interactive and customizable function based on TCGA and genotype tissue expression data. According to the median of HOXB4 gene expression, cut off-low (50%) and cut off-high (50%) values were used as the expression thresholds for dividing the patients into two groups, namely the low HOXB4 expression group and the high HOXB4 expression group.

### Cox Regression Analysis

To further evaluate the correlation of HOXB4 expression level with COAD, we employed univariate Cox regression analysis for calculating the association of the HOXB4 expression level with OS, disease-specific survival (DSS), and progression free interval (PFI) of the patients in two cohorts. We used the multivariate analysis to assess whether HOXB4 was an independent prognostic factor for CRC patient survival. The HOXB4 expression level was considered statistically significant in Cox regression analysis when *P*-value <0.05.

### Construction and Evaluation of Nomogram

For ascertaining the expression of HOXB4 in cancer prognosis, we built a nomogram which was considered as an effective and convenient method for predicting the OS, DSS, and PFI of CRC patients in the TCGA cohort (22, 23). The receiver operating characteristic (ROC) curve was performed to estimate the potential of HOXB4 as a prognostic marker in COAD. The whole data were filtered to remove missing and duplicated results, and transformed by log2 (TPM+1) using R packages of “rms” and “survival” in an R environment (R version: 3.6.3).

### Immune Infiltration Analysis

To determine the association between the HOXB4 expression level and immune infiltration according to the TCGA tumor data, we used the Tumor Immune Estimation Resource 2.0 (TIMER2.0) (http://cistrome.org/TIMER/) web server (24), and evaluated the correlation of the HOXB4 expression level with the abundance of infiltrating immune cells including B cells, CD4+ T cells, CD8+ T cells, macrophages, dendritic cells, neutrophils, and cancer-associated fibroblasts in CRC patients. In addition, we analysed the correlation between HOXB4 expression and tumor-infiltrating lymphocytes *via* the TISIDB database (http://cis.Hku.hk/TISIDB/) (25). The *P*-values and partial correlation (cor) values were calculated by means of the purity-adjusted Spearman’s rank correlation test.

### HOXB4-Related Gene Enrichment Analysis

We used Cistrome database (http://cistrome.org/db), which is a resource of human and mouse cis-regulatory information derived from DNase-seq, ChIP-seq, and ATAC-seq chromatin profiling assays, to obtain the information on gene regulatory analysis. Moreover, we overlapped the genes which were regulated by HOXB4 involved in CHEA Transcription Factor Targets Dataset and Cistrome cancer Dataset. Afterwards, we performed the R package of “cluster Profiler” to automate the results of the enrichment analysis (26). The *P* value <0.05 was deemed a statistically significant difference.

### Quantitative Real-Time PCR (qRT-PCR) and Western Blot Analysis

RNA was collected by the Trizol method, and 2 μg of RNA was used for cDNA synthesis with an RNA reverse transcription kit (TAKARA, Japan). The RT-qPCR kit was purchased from TAKARA, and RT-qPCR was performed with the CFX96 real-time system (Bio-Rad, USA). The primers were designed by Sangon Biotech Co., Ltd. (Shanghai, China), and the list of sequences is shown in **Table S1**.

### Western Blot Analysis

Proteins (30-50 μg) were separated on 12% sodium dodecyl sulfate polyacrylamide gels and were then transferred to polyvinylidene fluoride membranes (Micron Separations, Westborough, MA, USA) at 4°C. The membranes were blocked at 37°C for 1 h and were then incubated overnight with the primary antibodies at 4°C followed by incubation with HRP-conjugated secondary antibodies for 1 h at room temperature. The immunoreactive proteins were detected using the enhanced chemiluminescence system (Amersham-Pharmacia Biotech) and subsequent autoradiography.

### Cell Culture and MC-LR Treatment

The human colorectal adenocarcinoma cell line Caco2 was obtained from the American Type Culture Collection (Manassas, VA, USA) and synchronized by 24 h of serum deprivation. The cells were grown in DMEM with 10% fetal bovine serum (FBS) and antibiotics at 37°C in a humidified atmosphere of 5% CO_2_. The cells were treated with different doses of MC-LR (1, 5 and 10 µM) for 24, 48, or 72 h. The control cells were cultured with 0.1% DMSO.

### CCK-8 Assay

Cells were incubated in a 96-well plate with 3000 cells per well. Then, 10 μl of cell counting kit-8 reagent (Dojindo Inc., Kumamoto, Japan) was added to each well for 1 h, and the optical density (OD) value was detected using a Thermo Scientific Multiskan MK3 (Beijing, China).

### Wound Healing Assay

Cells were cultured in six-well plates until they reached 100% confluence. A vertical or horizontal wound was gently created in the monolayer using a 20 μl sterile pipette tip. The cells were then washed 3 times with growth medium to remove the detached cells, and fresh medium was added. The images were captured using an inverted microscope and camera at the designated times, and wound closure was assessed using Image Pro Plus 6.0. The rate of wound healing = [(the wound width of 0 h -t h)/0 h wound width] *100% (t: 24/48/72 h).

### Establishing Co-Culture System

Caco2 cells were pretreated with MC-LR at different concentrations (0, 1, 5, and 10μM) for 24 h in 24-well plates. Then, 600μL of supernatant was gathered and affiliated into the RAW264.7 cells. Besides, the cell proliferation reagent CCK-8 was applied to approximately measure the RAW264.7 cell viability.

To establish the co-culture system between Caco2 and RAW264.7 cells, 2 × 10^5^ RAW264.7 cells in a volume of 100μL were co-cultured in the upper chambers (8μm PET, Corning). Then, Caco2 cells were cultured in lower chambers and pretreated with MC-LR 5μM.

### Transwell Migration Assay

2 × 10^5^ cells were cultured in the 8 μm well (Coring, USA) for 24 hour and fixed in paraformaldehyde for 15 min, then stained with crystal violet for 10 min. Cells migrate through the well were counted in microscope.

### Flow Cytometry

Cells in each group were fixed in 75% ethanol for 24 h at -20°C. After treated with 50 μg/ml RNase (Sigma, USA) for 30 min at 37°C, the cells were stained with 50 μg/ml propidium iodide (Beyotime, Shanghai, China) for 15 min, then analyzed by BD Accuri c6 (BD Biosciences, USA), and Flow JO software.

### Statistics

Statistical analyses were performed with GraphPad Prism 8.0 (GraphPad Software, Inc.). For two-group comparisons, two-sided unpaired Student’s t-test was performed. For multiple comparisons, one-way followed by Dunnett’s *post hoc* test was performed. *P*-values < 0.05 were regarded as significant.

## Results

### Identifying the DEGs and Picking HOXB4

**Figure 1:**
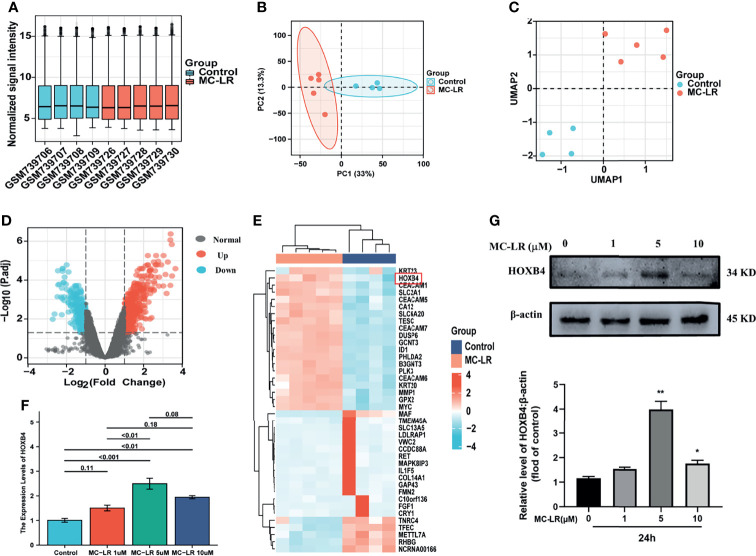
Identifying the DEGs and picking HOXB4. **(A)** Degree of normalization between samples analysis on base of GSE29861. **(B)** Principal component analysis for GSE29861. **(C)** Dimension reduction analysis for GSE29861. **(D)** Volcano plot of the 741 MC-LR-related DEGs. The significant DEGs with *P*<0.05 and [logFC]>1.0 were considered as the cut-off criteria. The red dots represent the significantly up-regulated genes and the blue dots indicate the significantly down-regulated genes. **(E)** Heatmap of the top high or low expression of 20 DEGs in MC-LR-treated and control samples. **(F)** RNA expression of HOXB4 in Caco2 cells treated with MC-LR for 24h n=3. **(G)** Representative immunoblot images and quantification of HOXB4 in Caco2 cells treated with MC-LR for 24h n=3. *P<0.05 and **P<0.01 vs. the untreated cells. DEGs, differentially expressed genes.

**Figures 1A–C** show a fine repeatability of data of GSE29861. Following the analysis of the GSE29861 dataset with R software, the difference of 20 DEGs between the MC-LR exposed Caco2 cell group and the control group was presented in volcano plot and heatmap (**Figures 1D, E**). Moreover, RT-qPCR and western-blots were used to confirm the finding that MC-LR exposure strongly increased HOXB4 expression in Caco2 cells (**Figures 1F, G**). Therefore, these results implied that HOXB4 may be the pivotal gene involved in MC-LR-mediated development of CRC cell proliferation and migration.

### HOXB4 Expression Analysis and Patient Characteristics

**Figure 2:**
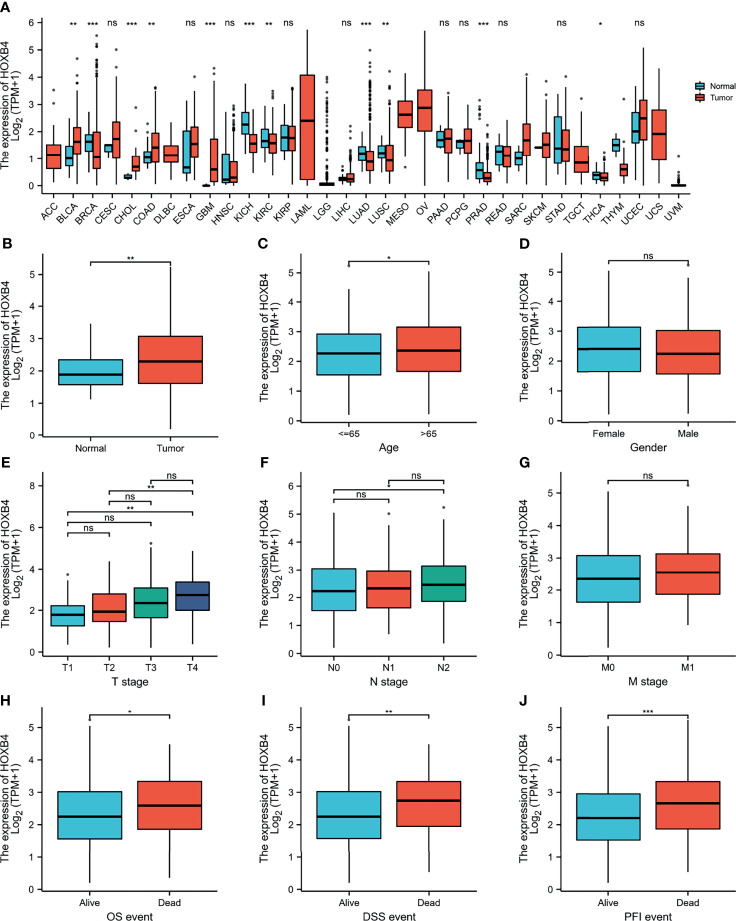
The mRNA expression levels of HOXB4 in cancers. **(A)** The HOXB4 mRNA expression in different cancers or in colon adenocarcinoma. **(B)** HOXB4 expression level in colorectal cancer tissue and normal tissue from TCGA dataset. **(C–G)** HOXB4 expression level in age, gender, different T stages, different N stages and different N stages in COAD. **(H–J)** HOXB4 expression level in COAD with OS event, DFS event, and PFI event. *P<0.05; **P<0.01; ***P<0.001 were considered statistically significant. OS, overall survival; DFS, disease-specific survival; PFI, progression free interval; COAD, colon adenocarcinoma; ns, no significance.

**Figure 3:**
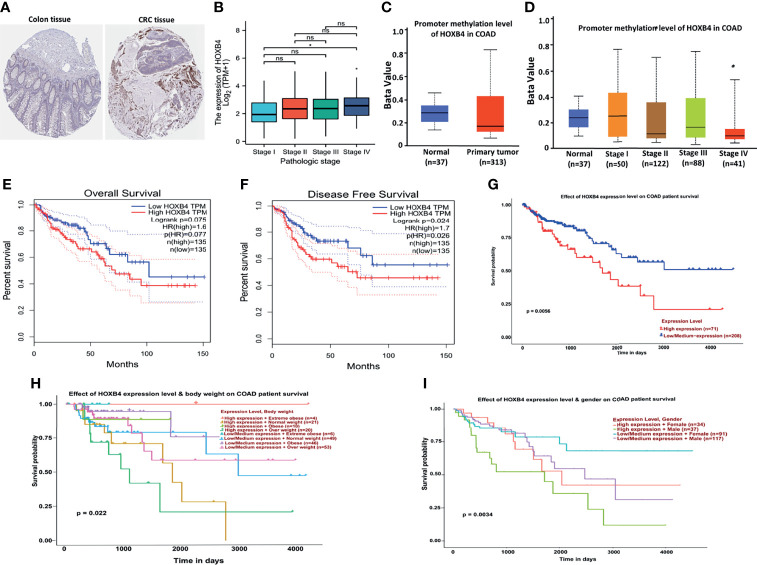
Correlation between HOXB4 and prognosis of colorectal cancer. **(A)** Protein expression of HOXB4 in COAD tissue and in normal tissue. **(B)** Correlation between the HOXB4 gene expression levels and the main pathological stages. **(C)** The promoter methylation level of HOXB4 in the primary tumor tissue and the normal tissue in COAD. **(D)** Low promoter methylation level of HOXB4 in pathological stage IV of COAD. **(E, F)** The HOXB4 gene expression-related overall survival and disease-specific survival of COAD tumor in TCGA. **(G–I)** Association between HOXB4 and survival of COAD patients. *P<0.05 was considered statistically significant. COAD, colon adenocarcinoma; ns, no significance.

To explore the oncogenic role of human HOXB4, we used the public database, TCGA, to analyze the mRNA expression of HOXB4 in various types of cancer. We find that the HOXB4 mRNA expression was significantly higher in tumor tissue of colon adenocarcinoma (COAD) (*P*=0.045) than in normal tissue in the TCGA (**Figure 2A**). A basic description of the RNAseq data and detailed clinical prognostic information of 480 COAD tumor samples and 41 normal tissue samples is shown in **Table S2**. Furthermore, we validated the HOXB4 mRNA expression in COAD and normal samples from TCGA (**Figure 2B**). Next, we analyzed the correlation of HOXB4 mRNA expression and clinicopathologic parameters in COAD patients (**Figures 2C–J**). The results indicated that no noteworthy difference was found in associations of HOXB4 mRNA expression levels with gender and pathologic metastasis stage (M stage), although the higher HOXB4 mRNA expression level was observed in patients in the advanced tumor stage (T stage) and lymph node stage (N stage) in OS, DSS and PFI events (**Figures 2E–J**). Correspondingly, Human Protein Atlas was used to evaluate the protein expression of HOXB4 in normal tissue and pathological tissue. As shown in **Figure 3A**, the HOXB4 protein expression is significantly up-regulated in COAD tissue compared with normal tissue (**Figure 3A**). Taken together, these results demonstrated that the augmentation of HOXB4 was highly related to the development of CRC.

### Correlation Between HOXB4 mRNA Expression and COAD Survival

To identify whether the up-regulation of HOXB4 was critical to promoting the progression of CRC and was employed as an important biomarker for clinical treatment, we performed the GEPIA to evaluate HOXB4 expression in different clinical stages of COAD. We observed that the expression of HOXB4 in stage IV was significantly higher than that in stage I (*P*=0.047) (**Figure 3B**). Surprisingly, the promoter methylation level of HOXB4 in COAD was also reduced in stage IV compared with that in stage I (*P*=0.046) (*P*<0.05, **Figure 3D**). However, the promoter methylation levels of HOXB4 in COAD between the primary tumor tissue and the normal tissue showed no difference (**Figure 3C**). Moreover, the correlation between HOXB4 levels and prognosis (OS HR=1.6, *P*=0.077; disease free survival [RFS] HR=1.7, *P*=0.024) of COAD presented patients categorized in the HOXB4-higher-score group had a significantly worse RFS (**Figures 3E, F**). Correspondingly, we applied the UALCAN to analyze the correlation of HOXB4 expression with the prognosis of patients with COAD. As shown in **Figures 3G–I**, highly expressed HOXB4 was linked to poor prognosis of COAD patient survival. The above results certified that HOXB4 expression impacted the prognosis of patients with COAD.

### Correlation Between Higher HOXB4 Expression and PFI in COAD *via* Univariate Analysis and Multivariate Analysis

**Figure 4:**
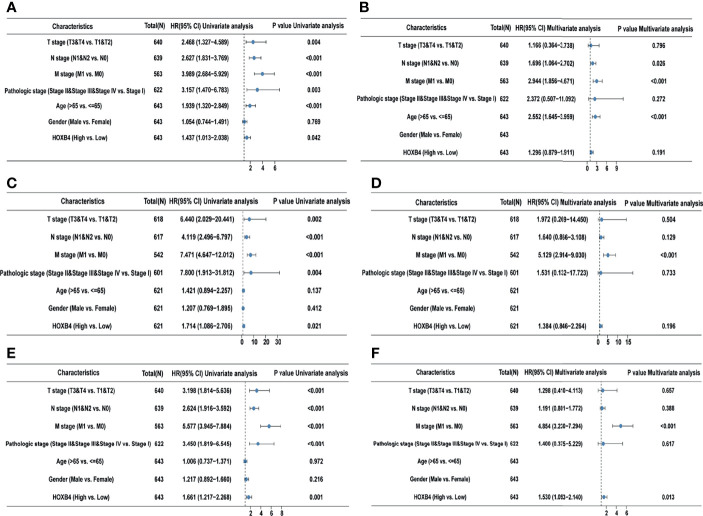
Univariate and multivariate regression analyses of HOXB4 expression and clinicopathologic parameters with OS event, DFS event, and PFI event in COAD patients. Univariate and multivariate regression analyses of HOXB4 expression and other clinicopathologic parameters with OS event **(A, B)**, DFS event **(C, D)** and PFI event **(E, F)** in COAD. OS, overall survival; DFS, disease-specific survival; PFI, progression free interval; COAD, colon adenocarcinoma.

According to the GEPIA and UALCAN, the higher HOXB4 mRNA expression displayed a poor survival of COAD. The high HOXB4 expression, pathologic grade and stage (TNM), pathologic stage, and age were negative predictors for OS and DSS in COAD patients with univariate analysis (**Figures 4A, C**). However, the multivariate analysis manifested that the high HOXB4 expression was not a significant factor for OS and DSS in COAD patients (**Figures 4B, D**). Interestingly, the high HOXB4 expression was a significantly negative predictor for PFI in COAD patients both in the test set (*P*=0.001) and the validation set (*P*=0.013) (**Figures 4E, F**). The relevant description is shown in **Table S3**.

### Nomogram Analysis of HOXB4 in COAD Prognosis

**Figure 5:**
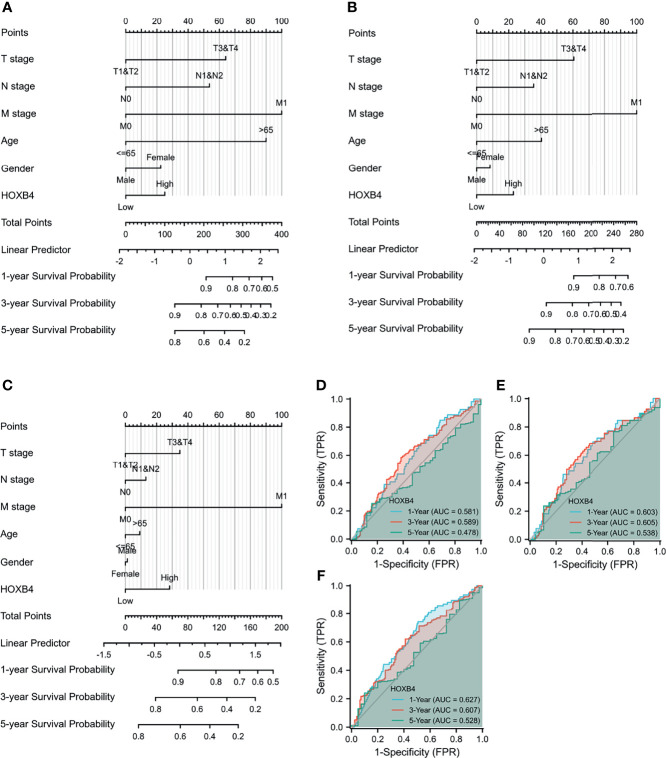
Evaluation of nomogram and receiver operating characteristic curves (ROC) analysis. **(A–C)** The nomogram analysis of HOXB4 TPM value, T stage, N stage, M stage, age, and gender. The total points projected on the bottom scales implied the probability of 1-, 3-, and 5-year OS event, DFS event, and PFI event. **(D–F)** ROC curves for the predictive efficiency of the HOXB4 for COAD patients based on TCGA cohorts. OS, overall survival; DFS, disease-specific survival; PFI, progression free interval; COAD, colon adenocarcinoma.

To further study the effect of HOXB4 combined with multiple indicators on cancer prognosis, we created a nomogram for predicting the OS, DSS, and PFI of COAD patients in the TCGA cohort. The T stage, N stage, M stage, age, gender, and HOXB4 were included as prognostic factors in the nomogram (**Figures 5A–C**). Subsequently, the time-dependent ROC curve analysis which was associated with the OS, DSS, and PFI was performed to measure the potential of HOXB4 as a prognostic marker in COAD. The areas under the curve (AUC) values for 1-, 3-, and 5-year OS were 0.581, 0.589, and 0.478, respectively; the AUC values for 1-, 3-, and 5-year DSS were 0.603, 0.605, and 0.538, respectively; the AUC values for 1-, 3-, and 5-year PFI were 0.627, 0.607, and 0.528, respectively (**Figures 5D–F**). The results demonstrated that HOXB4 had predictive power for 1-year survival of COAD patients. Additionally, the nomogram in which we included HOXB4 expression, clinical stage, age, and gender, had better predictive power for the prognostic factors including OS, DSS, and PFI of COAD patients, which might promote response evaluation and management of the patients.

### The Immune Infiltration Analysis Related to HOXB4

**Figure 6:**
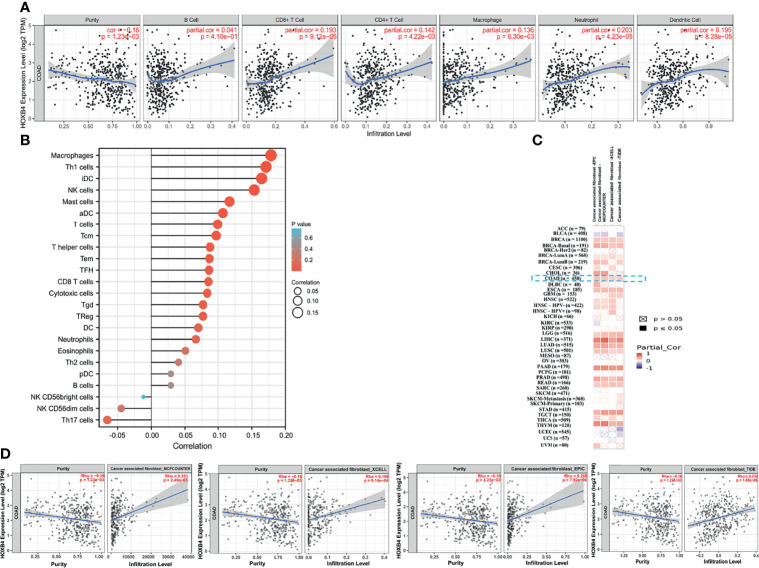
Correlation between HOXB4 expression level and the level of immune infiltration in COAD. **(A, B)** Correlation between HOXB4 expression and the infiltration level of different immune cells in colon adenocarcinoma with the TCGA. **(C, D)** Correlation between HOXB4 expression and immune infiltration of cancer-associated fibroblasts across all types of cancer in TCGA. COAD, colon adenocarcinoma.

Immune cell infiltration was considered as a remarkable component of the tumor microenvironment and was highly related to promoting the initiation, development or metastasis of cancer (27). We assessed the probable relationship between the HOXB4 expression and the infiltration stage of different immune cells in COAD from TCGA by using TIMER2.0, to find out whether HOXB4 regulates the tumor microenvironment. Previous studies have reported that cancer-associated fibroblasts in the stroma of the tumor microenvironment was closely link to the function modulation of all sorts of tumor-infiltrating immune cells (28, 29). Our findings implied that the expression level of HOXB4 had an apparently positive correlation with infiltrating levels of CD8+ T cells (r=0.193, *P*=9.12e-05), CD4+ T cells (r=0.142, *P*=4.22e-03), macrophages (r = 0.136, *P*=6.30e-03), neutrophils (r=0.203, *P*=4.23e-05), dendritic cells (r=0.195, *P*=8.28e-05) and tumor purity in COAD, but no association was observed with B cells (r=0.014, *P*=4.10e-01) (**Figures 6A, B**). Furthermore, we also observed a positive correlation of HOXB4 expression with the estimated infiltration value of cancer-associated fibroblasts in COAD (**Figures 6C, D**). The *P* value <0.05 was considered statistically significant.

### MC-LR Promotes High HOXB4 Expression and Alters the Cell Cycle by Augmenting C-myc Expression

**Figure 7:**
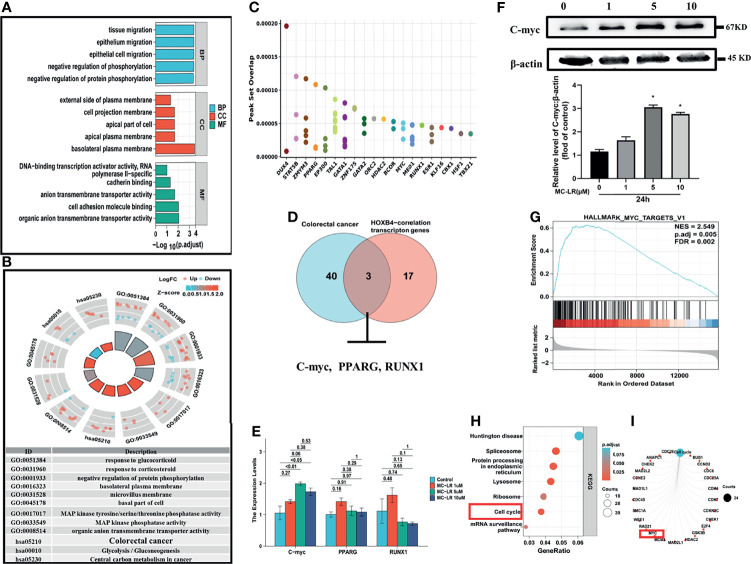
Cell cycle progression promoted by MC-LR-induced HOXB4 expression up-regulation. **(A)** GO enrichment analysis results of DEGs. **(B)** KEGG pathway analysis results of DEGs. **(C)** Top 20 genes overlapping in HOXB4 and the HOXB4-correction transcription genes by Cistrome. **(D)** Venn diagram of the genes involved in the colorectal cancer pathways and the top 20 genes in the HOXB4-correlation transcription genes. **(E)** mRNA expression levels of C-myc, PPARG and RUNX1 in Caco2 cells treated with 1, 5 and 10 μ M MC-LR for 24h n=3. **(F)** Representative immunoblot images and quantification of C-myc in Caco2 cells treated with MC-LR for 24h n=3. **(G)** Gene Set Enrichment Analysis (GSEA) for the potential altered pathways in the MC-LR-treated and Control groups. **(H, I)** KEGG enrichment analyses of the HOXB4-regulated genes. *P<0.05 vs. the untreated cells. DEGs, differentially expressed genes; GO, Gene Ontology; BP, biological process; CC, cellular component; MF, molecular function.

To elucidate the mechanisms of high HOXB4 expression induced by MC-LR, we firstly analyzed these DEGs between the MC-LR exposed Caco2 cell group and the control group by GO term enrichment analysis and KEGG pathway analysis using the R software. According to the results of GO analysis, the DEGs were classified into three categories: biological process (BP), cellular component (CC), and molecular function (MF), to show the main possible routes of the MC-LR in the Caco 2 cells. As shown in **Figure 7A**, in the BP group, the up-regulated genes were mainly enriched in activity regulation of protein phosphorylation. GO cell component analysis also revealed that the up-regulated DEGs were significantly enriched in basolateral plasma membrane. In addition, molecular function was mainly enriched in cell adhesion molecule binding (**Figure 7A**). **Figure 7B** show that the main enriched pathways of DEGs were mainly involved in CRC (**Figure 7B**). We employed the Cistrome to search out the master transcription-genes regulating HOXB4 in CRC cells, and obtained the top 100 transcription genes correlated with HOXB4 expression. Meanwhile, we overlapped HOXB4 with the HOXB4-correlation transcription genes, and showed the top 20 transcription genes in **Figure 7C**. Then, we overlapped the genes involved in the CRC pathways (**Figure 7D**) and the top 20 genes in the HOXB4-correlation transcription genes using the Funrich 3.0 (http://www.funrich.org/) (**Figure 7D**). RNA expression and immunoblot quantification analysis of PPARG, RUNX1 and C-myc in Caco2 cells treated with MC-LR for 24h were then preformed. The results suggested that C-myc instead of PPARG and RUNX1 positively correlated with HOXB4 expression (**Figures 7E, F**). Afterwards, we overlapped the genes which were regulated by HOXB4 between CHEA Transcription Factor Targets Dataset and Cistrome cancer Dataset. Subsequently, there were enriched by KEGG, and the results demonstrated that the most enriched term and pathway were related to the cell cycle (**Figures 7H, I**). Therefore, C-myc was assumed to be the master gene regulating HOXB4 expression and driving the cell cycle progression in MC-LR-mediated CRC progression.

### MC-LR Drives Cell Cycle Progression to Enhance Caco2 Cell Proliferation and Migration

**Figure 8:**
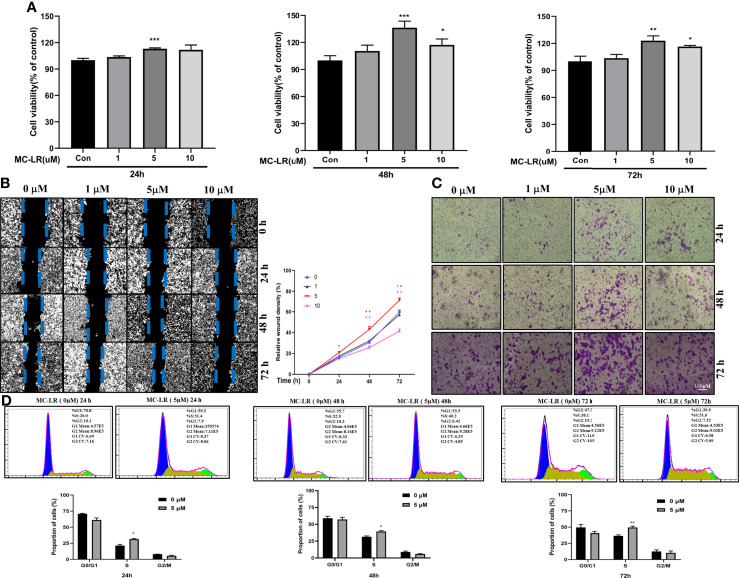
Caco2 cell proliferation, migration and cell cycle progression induced by MC-LR. **(A)** Cell viability of Caco-2 cells exposed to 1, 5, and 10 µM MC-LR for 24, 48, or 72 h. The control cells were cultured with 0.1% DMSO n=4. **(B)** The migration of Caco2 cells treated with 1, 5, and 10 µM MC-LR for 24, 48, or 72 h by Wound healing assay. n=3. **(C)** The images of the transwell assay results. Scale bar: 100 µm. **(D)** High cell proportion in S phase of the MC-LR-treated group. *P<0.05, **P<0.01 and ***P<0. 0.001 vs. the control group.

To verify the role of HOXB4 promoting CRC progression through regulating the cell cycle, we evaluated the biological effects of MC-LR on Caco2 cells *in vitro*. Caco2 cells were treated with various doses of MC-LR (1, 5 and 10 µM) for 24, 48, or 72 h. CCK-8 assay indicated that the 5 µM of MC-LR significantly promoted the proliferation of Caco2 cells after treatment for 24 h. Subsequently, the 10 µM of MC-LR also remarkably improved the proliferation of Caco2 cells after treatment for 48 h and 72 h (**Figure 8A**). Caco2 cells treated with 5 µM of MC-LR for 24 h, 48 h, and 72 h shown stronger migration capability than that in the control group (**Figure 8B**). Furthermore, transwell migration assay results indicated that 5 µM of MC-LR could strengthen the migration ability of Caco2 cells (**Figure 8C**). Cell cycle assay indicated that 5 µM of MC-LR significantly increased the S phase cell proportion in different time points (**Figure 8D**). These results demonstrated that the significant increase in the proportion of cells in S phase may play an important role in the MC-LR exposure-mediated enhancement of Caco2 cell proliferation.

### MC-LR-Induced Interaction Between Caco2 and Macrophages Promoted Macrophages Proliferation and Migration

**Figure 9:**
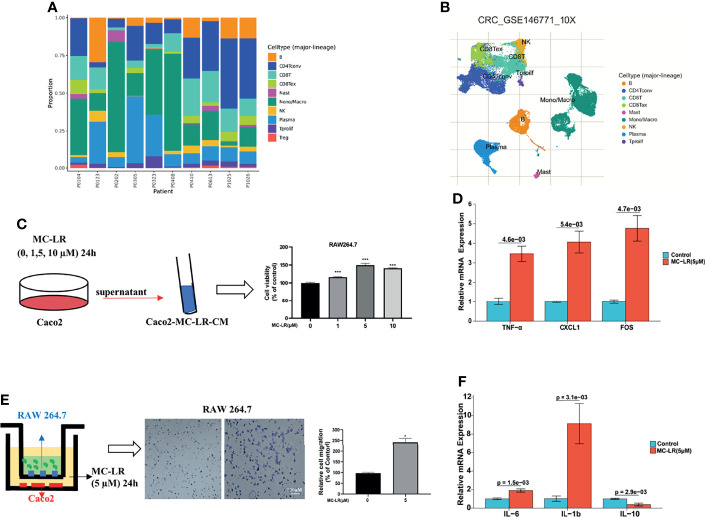
MC-LR-induced interaction between Caco2 and macrophages promoted macrophages proliferation and migration. **(A, B)** UMAP and bar charts reflected the cellular composition of the tumor microenvironment in CRC samples by TISCH. **(C)** The proliferation of RAW264.7 cells induced by MC-LR with CRC cells through CCK8 assay; ***P<0.001. **(D)** The migration of RAW264.7 cells were induced by MC-LR with CRC cells through Transwell assay; *P < 0.05. **(E)** Under the construction of the cell co-culture system for Caco2 and RAW264.7 cells, the mRNA levels of TNF-α, CXCL1, and FOS in Caco2 cells were measured by q-PCR; **(F)** Under the construction of the cell co-culture system for Caco2 and RAW264.7 cells, the mRNA levels of IL-6, IL-1b, and IL-10 in RAW264.7 cells were measured by q-PCR. All data are presented as the means ± SEM (n = 3).

Immune cells are critical components of the tumor microenvironment (TME). With aid of using TISCH (a comprehensive web resource enabling interactive single-cell transcriptome visualization of tumor microenvironment), the UMAP and bar charts reflected the cellular composition of the tumor microenvironment in CRC samples (**Figures 9A, B**). Then, we built a co-culture system to explore whether MC-LR exposure or HOXB4 upregulation to promote the macrophage infiltration of Colorectal cancer cell. Caco2 cells were pretreated with MC-LR at different concentrations (0, 1, 5, and 10μM) for 24 h in 24-well plates. Then, 600μL of supernatant was gathered and affiliated into the RAW264.7 cells. CCK8 assay indicated that supernatants including MC-LR treatment significantly increased the RAW264.7 cells proliferation (**Figure 9C**). Furthermore, transwell assay results indicated that 5 µM of MC-LR could strengthen the migration ability of RAW264.7 cells (**Figure 9D**). Under the exposure of MC-LR, Caco2 secreted a large abundant of inflammatory factors and cytokines (TNF-α, CXCL1and FOS) (**Figure 9E**), which produced a huge chemotactic effect on RAW264.7 cells and induced elevation of the surface M1-subtype biomarkers (IL-6 and IL1b) (**Figure 9F**). Together, these results suggested that MC-LR mediated interaction between Caco2 cells and macrophages induced macrophages proliferation and migration, and further might accelerated CRC progression.

## Discussion

MC-LR is universally considered to be a hepatotropic pollutant that noticeably exists in developing countries including China, where people are frequently exposed to this toxin directly through drinking water and indirectly through food chain. Previous observations *in vivo* and *in vitro* have proved that MC-LR induces obvious liver injury and stimulates hepatocarcinogenesis. However, these studies only focused on its toxicity in the liver tissue, and research on the gut as the primary exposed organ of MC-LR is rarely found. MCs, preferentially concentrating in the gastrointestinal tract, can exert gastrointestinal toxic effects (30). Besides, the previous studies *in vitro* by Ren, Y et al. (31) and Miao C et al. (32) reported that MC-LR exposure may boost migration and invasion of CRC cells. However, the potential molecular mechanism of how MC-LR stimulates the development of CRC is still unclear. Therefore, the effect of MC-LR exposure on gut injury currently becomes a crucial problem demanding prompt solutions. Through bioinformatics analysis, we ascertained, for the first time, that MC-LR promoted HOXB4 up-expression in CRC cells and regulated the pathways in cancer, that HOXB4 expression was highly-regulated in CRC patients and was positively correlated with the poor prognosis of CRC, that there was a statistically positive correlation between HOXB4 expression and the tumor-infiltrating immune cells. In addition, MC-LR promoted cell proliferation and augmented the expression of HOXB4 in Caco2 cells. These results may offer new evidence that MC-LR promotes CRC progression and that HOXB4 is a novel therapeutic target for CRC.

HOXB4 acts as a transcription factor to directly evoke or inhibit the expression of genes including Snail, Twist, and MMP3 involved in regulation of carcinogenesis and cancer metastasis (33–35). Some studies have revealed that HOXB4 reduces the cytotoxic effect of paclitaxel and cisplatin by up-regulating ABC transporters in ovarian cancer cells (36). The high expression of HOXB4 is positively correlated with poor prognosis of ovarian cancer (37). Meanwhile, HOXB4 transcription factors are potential targets and markers in malignant mesothelioma (37). However, how HOXB4 regulates the development of CRC is ambiguous. In an effort to identify the potential role of HOXB4 in CRC, we utilized the public database TCGA at first to verify the elevated expression levels of HOXB4 in colorectal tumors (**Figures 2A, B**). The HOXB4 mRNA expression level was observed to be higher in patients with advanced degree pathologic T and N stage in OS, DSS, and PFI events (**Figures 2E–J**). Then observed the higher expression of HOXB4 in pathological tissue compared with the normal tissue using Human Protein Atlas (**Figure 3A**). Besides, the HOXB4 mRNA expression level in stage IV was significantly higher than that in stage I (*P*=0.047) in COAD after the application of the GEPIA (**Figure 3B**). Surprisingly, we also discovered that the promoter methylation level of HOXB4 in COAD was reduced in stage IV compared with that in stage I (*P*=0.046) (*P <*0.05, **Figure 3D**). Furthermore, GEPIA and UALCAN were performed to prove the up-expression of HOXB4 in colorectal tumors, and the elevated expression of HOXB4 was observed to be significantly correlated with DFS in CRC patients, but no correlation with worse OS was observed (**Figures 3E, F**). This result was consistent with previous studies reporting that HOXB4 expression is regulated by promoter methylation (13, 38). Additionally, from the results of Cox regression analysis and nomogram, it was noticed that HOXB4 expression was an independent prognostic factor for COAD patients (**Figures 4**, **5**). Our study confirmed that HOXB4 was associated with poor prognosis of CRC, and expectedly, HOXB4 was a potential risk factor associated with the progression of CRC.

Considerable evidence indicates that MC-LR induces immunotoxicity in mammals through activating neutrophils and macrophages (39). Recently, sub-chronic MC-LR exposure has been reported to have dualistic immunomodulation effect on the innate-immune defense system in male zebrafish (40). MC-LR exposure induces chronic inflammatory response *via* MyD88-dependent toll-like receptor signaling pathway in male zebrafish. Adamovsky, O. et al. reported that MC-LR interferes with the potential of macrophage receptors and activates the NF-кB pathway to stimulate the production of pro-inflammatory cytokines (TNF-α and IL-6). In addition, HOXB4 is a key regulator of NK cell function, and its application in the generation of functional NK cells with increased lytic potential may be of great significance for cancer immunotherapy. We adopted multiple immune deconvolution methods to find a statistically positive correlation of HOXB4 expression with B cells, macrophages, CD8+ T cells, CD4+ T cells, dendritic cells, neutrophils, and cancer-associated fibroblasts (**Figures 6A, B**). Cancer-associated fibroblasts, along with immune cells, a variety of growth factors, chemokines, hormones and enzymes, form a complex network that regulates the tumor initiating cell growth in the tumor microenvironment, and thus are considered well-known effector cells in cancer immunotherapy (41). Furthermore, CD8+T cells are crucial effector cells in cancer immunotherapy (27). Intriguingly, HOXB4, which belongs to the HOX family, plays a crucial role in self-renewal of hematopoietic stem cells (36), and is involved in the regulation of T lymphocyte development (42). In addition, we found that macrophages were significantly infiltrated in CRC using TISCH (**Figures 9A, B**). Next, we built a co-culture system to explore the interaction between macrophages (RAW264.7) and Colorectal cancer cells (Caco2). The presence of MC-LR 5 µM MC-LR preconditioning was added in Caco2 for 24 h and the supernatant was collected. We found that MC-LR-induced interaction between Caco2 and macrophages promoted macrophages proliferation and migration under the exposure of supernatant (**Figures 9C, D**). Under the exposure of MC-LR, Caco2 secreted various inflammatory factors and cytokines (TNF-α, FOS, and CXCL1), which produced a huge chemotactic effect on RAW264.7 cells and induced elevation of the surface M1-subtype biomarkers (IL-6 and IL1b) (**Figure 9F**). Consistent with the previous researcher, they revealed that Macrophages, both M0 and M1 subtypes, were the most infiltrated immune cells in CRC tumor tissues compared with normal tissues (43). Together, these results further implied that HOXB4 was the key molecule for mediating MC-LR activation in the tumor immune infiltration and tumor microenvironment.

In order to explore the mechanism of MC-LR regulating HOXB4 high expression, we used the Cistrome database to search out the master genes regulating HOXB4 in CRC cells. Moreover, we overlapped the top 20 genes in the HOXB4-correlation transcription genes (**Figure 7C**). Analysis of enriched genes both in the MC-LR induced-pathways in cancer and the HOXB4-correlation transcription genes identified C-myc as a key target associated with HOXB4 high expression (**Figures 7E, F**). Notably, Lei-Lei Chen, et al. found that SNIP1 recruits TET2 to the promoters of c-MYC target genes, resulting in hypomethylation of the target gene promoter (44). Interesting, our results showed that the promoter methylation level of HOXB4 in COAD was also reduced in stage IV compared with that in stage I (**Figure 3D**). These results indicated that C-myc might regulated the methylation of HOXB4 promoter and led to HOXB4 high expression. Furthermore, we determined that the C-myc gene which was regulated by HOXB4 involved in increasing cell cycle progression (**Figures 7G–I**). Numerous evidence has revealed that C-myc dysregulation is a significant driver of colorectal carcinogenesis (45) and plays a vital role in colorectal cancer development (46, 47). Our finding revealed that MC-LR induced C-myc augmentation elevates the high expression of HOXB4 through increasing the cell cycle progression to enhance Caco2 cell proliferation.

Considerable evidence indicates that higher concentrations of MC-LR (from 1000 to 10 000 nM) for 24h were found to increase the expressions of multidrug resistance genes MRP1 and MDR1 in HepG2 cells (48). However, studies regarding the effects of MC-LR on the drug resistance in colorectal cancer cells are limited. In our study, Gene Set Enrichment Analysis (GSEA) analysis indicated that the C-myc (**Figure 7G**) signaling pathway was significantly enriched in the MC-LR-treated group. Notably, increasing evidences have indicated that the C-myc signaling pathway was involved in regulating chemoresistance in colorectal cancer. The C-myc/miR-27b-3p/ATG10 regulatory axis regulates chemoresistance in colorectal cancer (49). The study by Oh ET, et al. had reported that Brusatol-mediated inhibition of c-myc increases HIF-1α degradation and causes cell death in colorectal cancer under hypoxia (50). These results provide new evidence that MC-LR induced C-myc augmentation elevates the high expression of HOXB4 to enhance CRC patients gradually acquired rapid development of chemotherapy resistance. Hence, HOXB4/C-myc axis may be a novel therapeutic target for CRC patients treatment in the clinic in the MC-LR exposure.

However, our study has several limitations. First, bioinformatics analysis was not coupled with our own collected clinical sample. Second, we were not sure the interaction between the immune infiltration and CRC cells in the presence of MC-LR *in vivo*. Therefore, In the following future research, we would like to enlarge the sample size of CRC patients to provide a better understanding of the relationship between the exposure level of MC-LR and CRC. Meanwhile, studying the association of MC-LR exposure and HOXB4 levels with tumor stage and prognosis of CRC and exploring the underlying molecular mechanism would be beneficial as well. In addition, we will perform xenograft assay to demonstrate the underlying mechanisms by which MC-LR promotes colorectal cancer development, and further provide the clue in the status of immune infiltration to MC-LR-caused colorectal cancer progression.

In summary, we screened that the HOXB4 was a molecular target of MC-LR in promoting CRC progression with the aid of GEO. Our analyses showed statistically significant correlations of HOXB4 expression with prognosis, immune cell infiltration, constructing protein interaction networks and enrichment analysis of HOXB4-related transcription genes. Furthermore, we indicated the MC-LR induced C-myc augmentation elevates the high expression of HOXB4 through increasing the cell cycle progression to enhance Caco2 cell proliferation. These analysis results may aid the insight to the role of HOXB4 in MC-LR mediated-CRC progression from the perspective of clinical tumor sample studies.

## Data Availability Statement

The original contributions presented in the study are included in the article/**Supplementary Material**. Further inquiries can be directed to the corresponding author.

## Author Contributions

LW contributed to conception and design of the study, wrote the first draft of the manuscript. HJ organized the database. YZ wrote sections of the manuscript and organized the database. JW wrote sections of the manuscript. WC, YT, and KC organized the database and performed the statistical analysis. ZQ and WF performed the statistical analysis. ZZ contributed to conception and design of the study, administrated project, wrote review &edited. All authors contributed to manuscript revision, read, and approved the submitted version.

## Funding

This work was supported by the Natural Science Foundation of Chongqing (Grant No. cstc2019jcyj-msxmX0632) and the National Natural Science Foundation of China (Grant No. 81273156, 81302407 and 81803195).

## Conflict of Interest

The authors declare that the research was conducted in the absence of any commercial or financial relationships that could be construed as a potential conflict of interest.

## Publisher’s Note

All claims expressed in this article are solely those of the authors and do not necessarily represent those of their affiliated organizations, or those of the publisher, the editors and the reviewers. Any product that may be evaluated in this article, or claim that may be made by its manufacturer, is not guaranteed or endorsed by the publisher.
