# Supplementary material for: HOXB4 Mis-Regulation Induced by Microcystin-LR and Correlated With Immune Infiltration Is Unfavorable to Colorectal Cancer Prognosis
Source: Front Oncol. 2022 Feb 8;12:803493. doi: 10.3389/fonc.2022.803493 (PMC8861523; doi:10.3389/fonc.2022.803493)
Supplement: Supplementary file 1 [file Table_1.docx]

TABLE S1. Genes primers used in quantitative real-time PCR analyses.

| **Gene name** | | **Forward primer** |  |  | **Reverse primer** |  |  |
| --- | --- | --- | --- | --- | --- | --- | --- |
| Human | |  |  |  |  |  |  |
| HOXB4 |  | 5’-GCAGGTCTTGGAGCTGGAGAA-3’ | | | 5’-CTTGGTGTTGGGCAACTTGTG-3’ | | |
| C-myc |  | 5’-GGCTCCTGGCAAAAGGTCA-3’ | | | 5’-CTGCGTAGTTGTGCTGATGT -3’ | | |
| PPARG |  | 5’-GGGATCAGCTCCGTGGATCT-3’ | | | 5’- TGCACTTTGGTACTCTTGAAGTT-3’ | | |
| RUNX1 |  | 5’-GCCGAGTTTTCATCATTGCC-3’ | | | 5’- TGGAACGTCAGAGGAGAAACGA-3’ | | |
| GAPDH |  | 5’-TCAAGAAGGTGGTGAAGCAGG-3’ | | | 5’- AGCGTCAAAGGTGGAGGAGTG-3’ | | |
| CXCL1 |  | 5’-GGAAAGCTTGCCTCAATCCG -3’ | | | 5’- TGTTCCTATAAGGGCAGGGC-3’ | | |
| TNF-α |  | 5’-AGCCTGTAGCCCATGTTGTAG -3’ | | | 5’- GAGGAGCACATGGGTGGAG-3’ | | |
| FOS |  | 5’-CTTACTACCACTCACCCGCA -3’ | | | 5’- AGTGACCGTGGGAATGAAGT-3’ | | |
| β-actin |  | 5’-CCTTCCTGGGCATGGAGTC -3’ | | | 5’- TGATCTTCATTGTGCTGGGTG-3’ | | |
| Mouse |  |  | | |  | | |
| IL-6 |  | 5’-ATGAACTCCTTCTCCACAAGC -3’ | | | 5’-CTACATTTGCCGAAGAGCCCTCA -3’ | | |
| IL-1b |  | 5’-CCACAGACCTTCCAGGAGAATG-3’ | | | 5’-GTGCAGTTCAGTGATCGTACAGG-3’ | | |
| IL-10 |  | 5’-AGGGCACCCAGTCTGAGAACA-3’ | | | 5’-CGGCCTTGCTCTTGTTTTCAC -3’ | | |
| β-actin |  | 5’-AACAGTCCGCCTAGAAGCAC -3’ | | | 5’-CGTTGACATCCGTAAAGACC -3’ | | |
