# Supplementary material for: HOXB4 Mis-Regulation Induced by Microcystin-LR and Correlated With Immune Infiltration Is Unfavorable to Colorectal Cancer Prognosis
Source: Front Oncol. 2022 Feb 8;12:803493. doi: 10.3389/fonc.2022.803493 (PMC8861523; doi:10.3389/fonc.2022.803493)
Supplement: Supplementary file 2 [file Table_2.docx]

TABLE S2. The RNAseq data and detailed clinical prognostic information of different tumors in TCGA database.

| Group 1 | Group 2 | Number | Minimum value | Maximum value | Median | IQR | Lower quartile | Upper quartile | Mean | SD | SE |
| --- | --- | --- | --- | --- | --- | --- | --- | --- | --- | --- | --- |
| ACC | Tumor | 79 | 0.269 | 5.05 | 2.015 | 1.427 | 1.201 | 2.629 | 1.997 | 0.981 | 0.11 |
| BLCA | Normal | 19 | 0.627 | 3.87 | 1.957 | 1.142 | 1.412 | 2.554 | 2.034 | 0.898 | 0.206 |
| BLCA | Tumor | 414 | 0.187 | 6.286 | 2.728 | 1.217 | 2.16 | 3.377 | 2.766 | 0.971 | 0.048 |
| BRCA | Normal | 113 | 0.975 | 4.054 | 2.661 | 0.722 | 2.277 | 2.999 | 2.635 | 0.566 | 0.053 |
| BRCA | Tumor | 1109 | 0.046 | 6.779 | 1.966 | 1.83 | 1.338 | 3.169 | 2.286 | 1.276 | 0.038 |
| CESC | Normal | 3 | 2.114 | 2.8 | 2.658 | 0.343 | 2.386 | 2.729 | 2.524 | 0.362 | 0.209 |
| CESC | Tumor | 306 | 0.155 | 6.41 | 2.923 | 1.642 | 2.015 | 3.656 | 2.806 | 1.163 | 0.066 |
| CHOL | Normal | 9 | 0.439 | 0.91 | 0.602 | 0.275 | 0.567 | 0.842 | 0.681 | 0.187 | 0.062 |
| CHOL | Tumor | 36 | 0.469 | 4.081 | 1.392 | 0.843 | 1.137 | 1.98 | 1.6 | 0.774 | 0.129 |
| **COAD** | **Normal** | **41** | **1.338** | **3.459** | **1.872** | **0.603** | **1.602** | **2.205** | **1.968** | **0.515** | **0.08** |
| **COAD** | **Tumor** | **480** | **0.203** | **5.238** | **2.414** | **1.473** | **1.641** | **3.114** | **2.412** | **0.983** | **0.045** |
| DLBC | Tumor | 48 | 0.694 | 3.824 | 2.374 | 0.786 | 1.891 | 2.676 | 2.308 | 0.669 | 0.097 |
| ESCA | Normal | 11 | 0.636 | 4.197 | 1.431 | 1.121 | 1.05 | 2.171 | 1.792 | 1.147 | 0.346 |
| ESCA | Tumor | 162 | 0.514 | 4.591 | 2.514 | 1.386 | 1.782 | 3.168 | 2.472 | 0.912 | 0.072 |
| GBM | Normal | 5 | 0 | 0.045 | 0 | 0.022 | 0 | 0.022 | 0.013 | 0.02 | 0.009 |
| GBM | Tumor | 169 | 0.048 | 5.707 | 1.229 | 2.431 | 0.438 | 2.869 | 1.762 | 1.48 | 0.114 |
| HNSC | Normal | 44 | 0.072 | 4.051 | 0.491 | 1.768 | 0.264 | 2.032 | 1.096 | 1.117 | 0.168 |
| HNSC | Tumor | 502 | 0 | 4.261 | 0.683 | 1.384 | 0.313 | 1.697 | 1.081 | 0.997 | 0.045 |
| KICH | Normal | 24 | 2.217 | 5.161 | 3.552 | 0.956 | 2.986 | 3.942 | 3.487 | 0.697 | 0.142 |
| KICH | Tumor | 65 | 0.912 | 3.967 | 2.453 | 0.889 | 2.062 | 2.951 | 2.528 | 0.666 | 0.083 |
| KIRC | Normal | 72 | 1.511 | 4.169 | 2.614 | 0.937 | 2.233 | 3.17 | 2.747 | 0.679 | 0.08 |
| KIRC | Tumor | 539 | 0.624 | 4.863 | 2.606 | 0.89 | 2.142 | 3.032 | 2.581 | 0.687 | 0.03 |
| KIRP | Normal | 32 | 1.635 | 4.293 | 2.727 | 1.251 | 2.112 | 3.363 | 2.787 | 0.777 | 0.137 |
| KIRP | Tumor | 289 | 0.106 | 4.835 | 2.84 | 1.16 | 2.172 | 3.332 | 2.713 | 0.967 | 0.057 |
| LAML | Tumor | 151 | 0 | 6.556 | 3.442 | 4.593 | 0.523 | 5.116 | 3.074 | 2.159 | 0.176 |
| LGG | Tumor | 529 | 0 | 5.279 | 0.098 | 0.189 | 0.036 | 0.224 | 0.368 | 0.879 | 0.038 |
| LIHC | Normal | 50 | 0.086 | 1.366 | 0.56 | 0.229 | 0.456 | 0.685 | 0.579 | 0.242 | 0.034 |
| LIHC | Tumor | 374 | 0.024 | 3.653 | 0.575 | 0.585 | 0.338 | 0.923 | 0.71 | 0.507 | 0.026 |
| LUAD | Normal | 59 | 1.089 | 3.147 | 2.12 | 0.599 | 1.807 | 2.406 | 2.103 | 0.492 | 0.064 |
| LUAD | Tumor | 535 | 0.185 | 6.255 | 1.735 | 1.055 | 1.219 | 2.274 | 1.913 | 1.002 | 0.043 |
| LUSC | Normal | 49 | 0.995 | 3.018 | 2.109 | 0.636 | 1.932 | 2.567 | 2.172 | 0.461 | 0.066 |
| LUSC | Tumor | 502 | 0.207 | 5.56 | 1.824 | 1.388 | 1.217 | 2.604 | 1.962 | 0.962 | 0.043 |
| MESO | Tumor | 86 | 1.307 | 5.524 | 3.947 | 0.953 | 3.453 | 4.406 | 3.938 | 0.761 | 0.082 |
| OV | Tumor | 379 | 0.133 | 7.393 | 4.291 | 1.65 | 3.274 | 4.924 | 3.98 | 1.424 | 0.073 |
| PAAD | Normal | 4 | 2.577 | 3.413 | 2.932 | 0.345 | 2.775 | 3.121 | 2.964 | 0.351 | 0.176 |
| PAAD | Tumor | 178 | 0.346 | 4.551 | 2.862 | 1.076 | 2.198 | 3.274 | 2.76 | 0.774 | 0.058 |
| PCPG | Normal | 3 | 1.977 | 2.714 | 2.539 | 0.369 | 2.258 | 2.627 | 2.41 | 0.385 | 0.222 |
| PCPG | Tumor | 183 | 0.515 | 4.115 | 2.628 | 1.093 | 2.035 | 3.128 | 2.565 | 0.786 | 0.058 |
| PRAD | Normal | 52 | 0.2 | 4.176 | 1.213 | 0.971 | 0.669 | 1.64 | 1.442 | 1.061 | 0.147 |
| PRAD | Tumor | 499 | 0.04 | 3.432 | 0.671 | 0.66 | 0.416 | 1.076 | 0.821 | 0.572 | 0.026 |
| READ | Normal | 10 | 1.122 | 3.026 | 2.194 | 0.991 | 1.446 | 2.437 | 2.041 | 0.643 | 0.203 |
| READ | Tumor | 167 | 0.224 | 4.18 | 2.174 | 1.213 | 1.525 | 2.738 | 2.17 | 0.837 | 0.065 |
| SARC | Normal | 2 | 1.172 | 2.355 | 1.763 | 0.591 | 1.468 | 2.059 | 1.763 | 0.836 | 0.591 |
| SARC | Tumor | 263 | 0.105 | 6.056 | 2.881 | 1.347 | 2.182 | 3.529 | 2.772 | 1.173 | 0.072 |
| SKCM | Normal | 1 | 2.634 | 2.634 | 2.634 | 0 | 2.634 | 2.634 | 2.634 |  |  |
| SKCM | Tumor | 471 | 0.042 | 5.108 | 2.636 | 1.18 | 1.967 | 3.148 | 2.441 | 1.017 | 0.047 |
| STAD | Normal | 32 | 0.969 | 4.578 | 2.391 | 2.014 | 1.579 | 3.593 | 2.463 | 1.091 | 0.193 |
| STAD | Tumor | 375 | 0.065 | 5.525 | 2.29 | 1.465 | 1.674 | 3.139 | 2.436 | 0.971 | 0.05 |
| TGCT | Tumor | 156 | 0.128 | 4.479 | 1.722 | 1.433 | 1.174 | 2.607 | 1.889 | 0.921 | 0.074 |
| THCA | Normal | 58 | 0.167 | 1.732 | 0.89 | 0.52 | 0.577 | 1.097 | 0.871 | 0.353 | 0.046 |
| THCA | Tumor | 510 | 0.053 | 2.218 | 0.734 | 0.559 | 0.488 | 1.047 | 0.786 | 0.405 | 0.018 |
| THYM | Normal | 2 | 2.032 | 3.067 | 2.55 | 0.518 | 2.291 | 2.808 | 2.55 | 0.732 | 0.518 |
| THYM | Tumor | 119 | 0.245 | 4.701 | 1.435 | 0.821 | 0.963 | 1.785 | 1.411 | 0.608 | 0.056 |
| UCEC | Normal | 35 | 0.056 | 5.432 | 3.395 | 1.576 | 2.672 | 4.247 | 3.441 | 1.268 | 0.214 |
| UCEC | Tumor | 552 | 0 | 7.229 | 3.959 | 1.514 | 3.138 | 4.652 | 3.7 | 1.414 | 0.06 |
| UCS | Tumor | 56 | 0.324 | 5.922 | 3.339 | 2.286 | 1.977 | 4.263 | 3.151 | 1.501 | 0.201 |
| UVM | Tumor | 80 | 0 | 2.038 | 0.042 | 0.091 | 0.017 | 0.108 | 0.148 | 0.302 | 0.034 |
