# Supplementary material for: HOXB4 Mis-Regulation Induced by Microcystin-LR and Correlated With Immune Infiltration Is Unfavorable to Colorectal Cancer Prognosis
Source: Front Oncol. 2022 Feb 8;12:803493. doi: 10.3389/fonc.2022.803493 (PMC8861523; doi:10.3389/fonc.2022.803493)
Supplement: Supplementary file 3 [file Table_3.docx]

TABLE S3. The detailed clinical prognostic information of colorectal cancer in TCGA database.

| Characteristic | Low expression of HOXB4 | High expression of HOXB4 | *P* |
| --- | --- | --- | --- |
| n | 322 | 322 |  |
| T stage, n (%) |  |  | 0.006 |
| T1 | 15 (2.3%) | 5 (0.8%) |  |
| T2 | 64 (10%) | 47 (7.3%) |  |
| T3 | 212 (33.1%) | 224 (34.9%) |  |
| T4 | 28 (4.4%) | 46 (7.2%) |  |
| N stage, n (%) |  |  | 0.155 |
| N0 | 192 (30%) | 176 (27.5%) |  |
| N1 | 77 (12%) | 76 (11.9%) |  |
| N2 | 50 (7.8%) | 69 (10.8%) |  |
| M stage, n (%) |  |  | 0.715 |
| M0 | 232 (41.1%) | 243 (43.1%) |  |
| M1 | 41 (7.3%) | 48 (8.5%) |  |
| Pathologic stage, n (%) |  |  | 0.185 |
| Stage I | 65 (10.4%) | 46 (7.4%) |  |
| Stage II | 116 (18.6%) | 122 (19.6%) |  |
| Stage III | 88 (14.1%) | 96 (15.4%) |  |
| Stage IV | 40 (6.4%) | 50 (8%) |  |
| Primary therapy outcome, n (%) |  |  | 0.647 |
| PD | 15 (4.8%) | 18 (5.8%) |  |
| SD | 3 (1%) | 2 (0.6%) |  |
| PR | 6 (1.9%) | 10 (3.2%) |  |
| CR | 134 (42.9%) | 124 (39.7%) |  |
| Gender, n (%) |  |  | 0.155 |
| Female | 141 (21.9%) | 160 (24.8%) |  |
| Male | 181 (28.1%) | 162 (25.2%) |  |
| Race, n (%) |  |  | 0.335 |
| Asian | 5 (1.3%) | 7 (1.8%) |  |
| Black or African American | 30 (7.6%) | 39 (9.9%) |  |
| White | 164 (41.6%) | 149 (37.8%) |  |
| PFI event, n (%) |  |  | 0.011 |
| Alive | 254 (39.4%) | 225 (34.9%) |  |
| Dead | 68 (10.6%) | 97 (15.1%) |  |
| DSS event, n (%) |  |  | 0.034 |
| Alive | 283 (45.5%) | 261 (42%) |  |
| Dead | 30 (4.8%) | 48 (7.7%) |  |
| OS event, n (%) |  |  | 0.076 |
| Alive | 267 (41.5%) | 248 (38.5%) |  |
| Dead | 55 (8.5%) | 74 (11.5%) |  |
| Neoplasm type, n (%) |  |  | 0.009 |
| Colon adenocarcinoma | 224 (34.8%) | 254 (39.4%) |  |
| Rectum adenocarcinoma | 98 (15.2%) | 68 (10.6%) |  |
| Colon polyps present, n (%) |  |  | 0.576 |
| No | 113 (35%) | 111 (34.4%) |  |
| Yes | 54 (16.7%) | 45 (13.9%) |  |
| History of colon polyps, n (%) |  |  | 0.303 |
| No | 195 (35.1%) | 182 (32.8%) |  |
| Yes | 83 (15%) | 95 (17.1%) |  |
| Lymphatic invasion, n (%) |  |  | 0.906 |
| No | 178 (30.6%) | 172 (29.6%) |  |
| Yes | 116 (19.9%) | 116 (19.9%) |  |
| Age, n (%) |  |  | 0.094 |
| <=65 | 149 (23.1%) | 127 (19.7%) |  |
| >65 | 173 (26.9%) | 195 (30.3%) |  |
| Weight, n (%) |  |  | 0.064 |
| <=90 | 115 (33%) | 129 (37.1%) |  |
| >90 | 61 (17.5%) | 43 (12.4%) |  |
| Height, n (%) |  |  | 0.621 |
| <170 | 77 (23.4%) | 82 (24.9%) |  |
| >=170 | 88 (26.7%) | 82 (24.9%) |  |
| BMI, n (%) |  |  | 0.055 |
| <25 | 45 (13.7%) | 62 (18.8%) |  |
| >=25 | 120 (36.5%) | 102 (31%) |  |
| Residual tumor, n (%) |  |  | 0.743 |
| R0 | 234 (45.9%) | 234 (45.9%) |  |
| R1 | 2 (0.4%) | 4 (0.8%) |  |
| R2 | 19 (3.7%) | 17 (3.3%) |  |
| CEA level, n (%) |  |  | 0.659 |
| <=5 | 136 (32.8%) | 125 (30.1%) |  |
| >5 | 76 (18.3%) | 78 (18.8%) |  |
| Perineural invasion, n (%) |  |  | 0.921 |
| No | 93 (39.6%) | 82 (34.9%) |  |
| Yes | 33 (14%) | 27 (11.5%) |  |
| Age, meidan (IQR) | 67 (57, 74) | 69 (60, 77) | 0.015 |
